# Evaluation of a vaccine candidate isolated from *Cryptosporidium parvum* oocyst in mice

**DOI:** 10.14202/vetworld.2022.2772-2784

**Published:** 2022-12-05

**Authors:** Dina Aboelsoued, Hend H. A. M. Abdullah, Kadria N. Abdel Megeed, Soad E. Hassan, Nagwa I. Toaleb

**Affiliations:** Department of Parasitology and Animal Diseases, Veterinary Research Institute, National Research Centre, Dokki, Giza, Egypt

**Keywords:** affinity chromatography, *Cryptosporidium parvum*, cytokines, enzyme-linked immunosorbent assay, histopathology, polymerase chain reaction, vaccine

## Abstract

**Background and Aim:**

Cryptosporidiosis is a leading cause of diarrheal disease worldwide and is an animal and public health burden. This study aimed to evaluate the protective potential of affinity-purified *Cryptosporidium parvum* oocyst antigen as a vaccine candidate according to fecal oocyst shedding, humoral and cellular immune responses, histopathological changes, and the number of parasite developmental stages in ileal and hepatic tissues.

**Materials and Methods:**

We isolated oocysts from naturally infected buffalo calves and identified them molecularly as *C. parvum* isolates (GenBank: ON730707 and ON730708) by targeting the *Cryptosporidium* oocyst wall protein gene. We propagated the *C. parvum* oocysts in mice. In addition, we prepared crude antigen from the isolated oocysts by purification using cyanogen bromide-activated Sepharose-4B affinity chromatography coupled with rabbit hyperimmune serum. Then, we divided 81 parasite-free mice into three groups: (1) non-vaccinated non-infected mice, (2) mice orally infected with 1 × 10^5^ *C. parvum* oocysts on week 4 of the experiment, and (3) mice immunized twice with 40 μg/kg of the purified fraction at 2-week intervals. Then, we challenged the vaccinated group with *C. parvum* oocysts after 2 weeks, and the positive control group was infected at the same time.

**Results:**

We observed a prolonged prepatent period and decreased oocyst shedding in the vaccinated infected mice compared with the non-vaccinated infected mice (t < 0.001). The vaccinated mice had significantly higher immunoglobulin G levels than those in the other two groups at all examined weeks. In addition, the production of cytokines interferon-gamma, interleukin (IL)-10, IL-12, and IL-15 was activated post-vaccination. After the challenge, all tested cytokines were significantly increased (p < 0.001) in the two infected groups compared with the non-vaccinated non-infected group, with the highest levels in the vaccinated infected group. Vaccinated infected mice exhibited significantly fewer pathological lesions in the ileum and liver than non-vaccinated infected mice, which showed prominent histopathological lesions. Endogenous developmental stages of *C. parvum* indicated that the ileum was more parasitized than the liver and that vaccination resulted in a lower number of oocysts in ileal and hepatic tissues (p < 0.05).

**Conclusion:**

Our prepared affinity-purified vaccine candidate could be promising in protecting against cryptosporidiosis.

## Introduction

*Cryptosporidium* spp. are the leading cause of protozoal diarrhea in humans and animals [1]. Zoonotic cryptosporidiosis occurs globally, particularly in developing countries with poor sanitary conditions [2]. Although cryptosporidiosis tends to cause self-limiting diarrhea in immunocompetent humans, it is the second major cause of infant diarrhea and death, after rotavirus, in Africa and Asia [3]. The *Cryptosporidium* parasite infects a variety of mammals, including bovines (especially young calves), dogs, cats, birds, and rabbits [4]. Bovine cryptosporidiosis caused by *Cryptosporidium parvum* is a critical problem in the dairy industry, especially in newborn calves, where the infection is life-threatening [5], and *C. parvum* was reported as the most dominant *Cryptosporidium* species in farm animals [6]. Moreover, bovine cryptosporidiosis is a public health concern because *C. parvum* is a zoonosis [7]. Even though there is extensive research on drug treatments against *Cryptosporidium* spp., effective curative treatments remain scarce [8]. Moreover, despite the urgent need, there are no commercial vaccines available to protect humans or animals from cryptosporidiosis [9, 10]. Failure to develop an efficient protective vaccine against cryptosporidiosis is mostly due to its life cycle [11].

Control of parasitic infections depends on the production of multiple cytokines to activate the host defense mechanisms that limit parasite invasion, survival, and reproduction [12]. *Cryptosporidium parvum* develops in the apical portion of intestinal epithelial cells, and systemic infection or deep tissue penetration has never been reported [13, 14]. It is considered an invasive mucosal pathogen and elicits a strong cell-mediated response following both primary and secondary infections [13, 15]. In addition, cryptosporidiosis may extend to the biliary tract epithelium. The free stages of the parasite are released into the lumen at different phases of its life cycle, where they infect other host epithelial cells. During replication within these cells, it uses the host’s biological processes to its benefit, and the infected host epithelial cells communicate with the immune system by activating acute mucosal inflammatory reactions as a response [16]. Thus, the epithelial cells play a key role in both parasite replication and protective immune responses [13]. Host resistance after *C. parvum* infection involves both innate and adaptive immune responses [17, 18]. Early in *Cryptosporidium* infection, the main cytokine involved in the coordination of innate and adaptive immune responses is interferon-gamma (IFN-γ), which is secreted by natural killer (NK) cells, dendritic cells, and macrophages [19]. Natural killer cells are a major source of IFN-γ in early infection [19] and produce high levels in response to induction by the T-helper 1 (Th1) cytokines interleukin (IL)-12, IL-15, and IL-18 [20, 21], which help to control *C. parvum* [22, 23]. Natural killer cells, activated by IL-15 secreted by intestinal epithelial cells [24], have been reported to lyse *C. parvum*-infected epithelial cells *in vitro* [25]. Early innate immune protection mediators involve the small intestine mucus layer, intestinal epithelial cells, cytokines, chemokines, and antimicrobial peptides [11, 20]. Interleukin-12 is a key in the early protective immune response, upregulating IFN-γ production by NK cells and T cells [26] by boosting the Th1 pathway [27, 28] and indirectly stimulating the antiparasitic and antimicrobial activity of macrophages [29]. In addition, mice with impaired IL-12 function have been reported as susceptible to cryptosporidiosis [27]. Interleukin-10 is secreted by many cells in the intestinal mucosa and provokes mucosal inflammatory and immune responses [30]. It may also protect against excessive host-threatening immune responses during infection [31].

There are no appropriate therapies for clearing *C. parvum*. Thus, the use of alternative immunotherapies or the establishment of a vaccine candidate containing *Cryptosporidium* parasites that are incapable of causing disease would be incredibly helpful in providing protection, decreasing infection severity, or decreasing the longevity of cryptosporidiosis [20]. Hence, our study evaluated the protective potential of *C. parvum* affinity-purified protein as a vaccine candidate in experimentally challenged mice.

## Materials and Methods

### Ethical statement

All experimental procedures were conducted according to the guidelines of the International Animal Ethics Committee and the institutional guidelines of the National Research Centre (NRC) Animal Research Committee (protocol no. 19–152).

### Study period and location

This study was a cross-sectional study conducted from January 2022 to June 2022 in NRC, Giza, Egypt. Fecal samples were collected from buffalo calves reared by local farmers in Giza (29° 58’ 27.00” N, 31° 08’ 2.21” E) and Beni-Suef (29° 03’ 60.00” N, 31° 04’ 60.00” E) Governorates, Egypt. In addition, an experimental study including infection and antibody preparation was performed at the Animal House, NRC, Egypt.

### Animals

#### Buffalo calves

We collected 20 fecal samples from newborn buffalo calves aged 5–20 days that were suffering from severe mucoid diarrhea and were reared by local farmers in Giza and Beni-Suef Governorates, Egypt. The samples were collected directly from the calves’ rectum into plastic cups using sterile plastic gloves and transported in an icebox (4°C) to the laboratory for examination on the day of collection.

#### Mice and rabbits

We obtained 90 newborn male laboratory-bred Swiss albino mice (weight: 20–25 g) from the Animal House, NRC, Egypt, and five New Zealand male rabbits (6 weeks of age, 1.5–2 kg each) from the local market for use in this study. The animals were confirmed as parasite-free for 3 consecutive days before commencing experiments involving sedimentation, flotation [32], and modified Ziehl–Neelsen (MZN) staining [33]. Strict sanitized conditions were maintained, and all mice were given access to food and water *ad libitum*. The animals were allowed to adapt to the experimental conditions for 1 week.

### Parasites

Each collected calf fecal sample underwent MZN staining [33] and was then examined for the presence of *Cryptosporidium* using light microscopy (Olympus Corporation CX41, Japan) with an oil immersion objective. The *Cryptosporidium* oocysts present in positive samples were concentrated by flotation using Sheather sugar solution [34] and preserved in potassium dichromate solution (K_2_Cr_2_O_7_, 2.5%) at 4°C. The oocysts were washed in phosphate-buffered saline (PBS; 0.01 M, pH 7.2) before being used to infect mice experimentally.

### Genomic DNA extraction

DNA was extracted from *Cryptosporidium*-positive fecal samples (as identified by fecal microscopical examination) using a QIAamp DNA Stool Mini Kit (Qiagen Inc., USA) according to the manufacturer’s protocol after subjecting the samples to five freezing (in liquid nitrogen) and thawing (in a water bath at 95°C) cycles. After extraction, the DNA concentration in each sample was measured using a Q9000 microvolume spectrophotometer (Quawell, USA), and then the samples were stored at −20°C for molecular investigation.

### Polymerase chain reaction (PCR) for *Cryptosporidium* identification and sequencing

The extracted DNA was screened using a specific primer pair targeting 553 bp of the *Cryptosporidium* oocyst wall protein (COWP) gene (Cry9: Forward 5'-GGACTGAAATACAGGCATTATCTTG-3' and Cry15: Reverse 5'- GTAGATAATGGAAGAGATTGTG-3') [35]. Each PCR reaction was performed in a final volume of 25 μL using 2× OnePCR master mix solution (GeneDireX, Taiwan) as per the manufacturer’s protocol. Amplification was performed out on a thermal cycler (Bio-Rad Laboratories, Singapore) as follows: Initial denaturation of 94°C for 5 min; 35 cycles including denaturation at 94°C for 30 s, annealing at 55°C for 40 s, and extension at 72°C for 45 s; and final extension at 72°C for 10 min. Then, the amplified products underwent electrophoresis on a 1.5% agarose gel stained with RedSafe (Intron Biotechnology, Republic of Korea) and were visualized using a Molecular Imager (Bio-Rad Laboratories). The fragment sizes were estimated using an H3 RTU 100 bp DNA ladder (GeneDireX).

We used a QIAquick Gel Extraction Kit (Qiagen) to purify the PCR products positive for *Cryptosporidium* as per the manufacturer’s protocol. Then, the purified PCR products were sequenced on an ABI 3130 automated sequencer (Applied Biosystems, USA) using a Big Dye Terminator v3.1 Cycle Sequencing Kit (Applied Biosystems). The obtained sequences were assembled and corrected using ChromasPro software (ChromasPro 1.7, Technelysium Pty Ltd., Australia). The corrected sequences were compared with those available in GenBank using BLAST (https://blast.ncbi.nlm.nih.gov/Blast.cgi) and submitted to GenBank.

### Phylogenetic analysis

Multiple sequence alignment was performed using CLUSTAL W v1.83, as designed by Thompson *et al*. [36], in the MegAlign module of the Lasergene software package (DNASTAR, USA). Phylogenetic analyses were performed using maximum likelihood, neighbor-joining, and maximum parsimony analyses in MEGA6 [37].

### Oocysts propagation

We used nine 3-week-old parasite-free Swiss albino mice for the production of oocysts for antigen preparation. They were experimentally infected using gastric tubes with a single dose of 1 × 10^5^ *Cryptosporidium* oocysts 1 h before a meal. The oocysts were collected from naturally exposed buffalo calves [38]. After 4 days, we collected the fecal pellets of the infected mice every day for 3 weeks and examined them using MZN staining [33]. If oocysts were present in fecal pellets, they were concentrated by centrifugation (500× *g*/30 min/4°C) in Sheather sugar solution [39].

### Antigen preparation

*Cryptosporidium* oocyst antigen was prepared by 20 cycles of repeated freezing and thawing, 12 cycles of sonication for 30 s (Sonics Vibra Cell VCX750; Sonics & Materials, Inc., USA), and centrifugation at 12,000× *g* for 15 min at 4°C according to Kaushik *et al*. [40].

### Rabbit hyperimmune serum preparation

We raised hyperimmune serum against oocyst antigen in five rabbits according to the method of Fagbemi *et al*. [41]. In brief, 40 μg/kg of *C. parvum* oocyst antigen was mixed with an equal volume of Freund’s complete adjuvant and injected subcutaneously into each of 5 rabbits. Two weeks later, a booster dose of 40 μg/kg of antigen in Freund’s incomplete adjuvant was injected subcutaneously. The second and third booster doses were injected on days 21 and 28, respectively. We collected blood samples from rabbit’s ear vein 4 days after the third booster dose. The prepared hyperimmune serum was stored at −20°C until use.

### Affinity purification of *Cryptosporidium* oocyst antigen

We dialyzed the rabbit hyperimmune serum for 3 days in coupling buffer (0.1 M NaHCO_3_, 0.5 M NaCl, pH 8.4) and then coupled it to cyanogen bromide-activated Sepharose-4B (Sigma-Aldrich, USA) at a ratio of 2 mg/mL swollen beads, according to manufacturer’s instructions. Then, the antigen was applied to a Flex-Column chromatography column (Kimble Chase Life Science and Research Products, LLC, USA), and the bound fractions were eluted using 50 mM glycine containing 500 mM NaCl (pH 2.3). The purified fraction was stored at −20°C until use. The protein concentrations of the crude and the affinity-purified antigen were estimated as described by Lowry *et al*. [42].

### Vaccination protocol in mice

We randomly divided the mice into three groups (n = 27/group). The first group comprised non-vaccinated non-infected mice (negative control). The mice in the second group were orally infected using gastric tubes with 1 × 10^5^ *C. parvum* oocysts in 250 μL PBS (pH = 7.2) [38] 1 h before a meal on week 4 of the experiment (positive control). The mice in the third group were immunized with the *Cryptosporidium*-specific purified fraction by two doses of subcutaneous injection at 2-week intervals. The vaccine comprised 40 μg/kg of purified fraction mixed with 250 μL PBS (pH = 7.2) and was emulsified with an equivalent volume of complete Freund adjuvant (Sigma-Aldrich) in the first dose and incomplete Freund adjuvant (Sigma-Aldrich) in the second dose [28]. Then, the vaccinated mice were orally challenged with *C. parvum* oocysts after 2 weeks, and the positive control group was infected at the same time. Three mice per group were sacrificed each week, and blood samples were collected from all groups before immunization (day 0) until the 7^th^ week post-first vaccination dose. The sera were separated and frozen at −20°C for further use. From day 3 post-infection (PI), we collected mice fecal pellets every day for 3 weeks to monitor the oocyst count throughout the experiment. On day 10 PI, we sacrificed three mice from each group to examine histopathological changes in tissues.

### Evaluation of the protective value of the prepared *Cryptosporidium* vaccine candidate

#### Oocyst shedding

Around 20 mg of fecal pellets were collected daily from each mouse in the vaccinated and non-vaccinated infected groups until the end of the experiment. Each fecal sample was smeared on a glass slide and stained using the MZN technique [33]. The number of oocysts was counted in 50 microscopic fields at 1000× magnification (Olympus Corporation CX41, Japan) [43]. Fecal pellets were also collected from the non-vaccinated non-infected mice group in parallel to the infected groups and examined to confirm their negativity throughout the experiment.

#### Humoral immune response

The humoral response (serum immunoglobulin G [IgG] response) evoked by vaccination and infection was assessed by indirect enzyme-linked immunosorbent assay (ELISA) to evaluate the success of the affinity-purified fraction in achieving protective activity against cryptosporidiosis. Immunoglobulin G levels were measured in mice sera collected at weekly intervals along the experiment time, as previously described by Priest *et al*. [44] and Liu *et al*. [28]. Briefly, flat-bottom 96-well ELISA plates were coated with 4 μg/mL of purified fraction in carbonate buffer. Serum samples from mice in the three groups were diluted to 1:100 and added to the plates separately in triplicate. Then, we added antimouse IgG horseradish peroxidase-labeled conjugate (1:1000; Sigma-Aldrich) and ortho-phenylenediamine (Sigma-Aldrich) substrate buffer (1 mg/mL). The optimum antigen concentration and antibody and conjugate dilutions were determined by checkerboard titration. The plates were read spectrophotometrically at 450 nm using an ELx800UV microplate reader (BioTek Instruments, Inc., USA).

#### Cellular immune response

We measured the levels of IFN-γ, IL-10, IL-12, and IL-15 in the mice sera (collected each week from the non-vaccinated and vaccinated groups) using sandwich ELISA kits (Sunlong Biotech Co., Ltd., China) as described by the manufacturer. Optical density (OD) was measured at 450 nm using a microplate reader (BioTek Instruments), and concentrations were calculated using standard curves that were performed for the same assays.

#### Histopathological examination

On day 10 PI, we sacrificed three mice from each group and collected the ileal and hepatic tissues for histopathological examination. The samples were prepared as per Bancroft and Stevens [45]. Then, the blocks of paraffin-embedded tissue were cut into 4–6 μm-thick sections using a microtome and stained with hematoxylin and eosin to study tissue structure and assess the infection progression using light microscopy.

#### Estimation of Cryptosporidium developmental stages and oocyst count in ileal and hepatic sections

The *Cryptosporidium* endogenous developmental stages count was calculated in ileal and hepatic tissue sections as the number of oocysts per cross-sectional area using a QWin DW3000 image analysis system (LEICA Imaging Systems Ltd., England). We assessed six of the most representative fields for each section in all groups at 400× magnification.

### Statistical analysis

Statistical analysis was performed using a statistical package for the social sciences version 19.0 for Windows (IBM Corp., NY, USA), and values were presented as the mean and standard error. Data for the oocyst count in feces and histopathological sections, ELISA, and cytokine concentrations were analyzed using Kolmogorov–Smirnov test of normality, which indicated that the most data were normally distributed (parametric data). Thus, descriptive analyses, t-tests, one-way analysis of variances, and *post hoc* tests were performed for intergroup comparisons. p < 0.05 was considered statistically significant.

## Results

### Polymerase chain reaction and phylogenetic analysis

**Figure-1:**
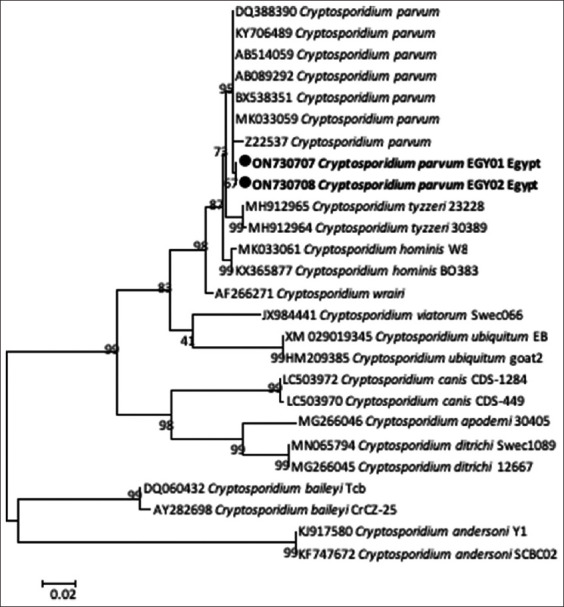
Phylogenetic analysis using the maximum likelihood method based on *Cryptosporidium* oocyst wall protein gene for *Cryptosporidium* spp. The new obtained sequences in this study are highlighted (black dots). There were a total of 495 positions in the final dataset. The scale bar represents a 2% nucleotide sequence divergence.

*Cryptosporidium* DNA was detected in the fecal samples of 15 out of 20 buffalo calves (infection rate, 75%) using the COWP gene. A BLAST search revealed two genotypes of *C. parvum* in the investigated calves, and we deposited the sequences in GenBank (ON730707, 10 calves; ON730708, five calves). These two genotypes were 99.8% (594/595) identical to those of *C. parvum* detected in humans from Egypt (GenBank: MK033059). Phylogenetic analysis revealed that these genotypes clustered in a well-supported branch (bootstrap value, 96%) with other *C. parvum* sequences (Figure-1).

### Identification of purified vaccine candidate

The affinity purification of crude *C. parvum* oocyst antigen resulted in one purified specific fraction (vaccine candidate) that represented 1.25% of the total protein. After the crude antigen was applied to the column, it possessed 72.64% of the initial antigenic activities of the crude extract. The vaccine candidate was then characterized by sodium dodecyl sulfate-polyacrylamide gel electrophoresis, western blot, and amino acid analysis, and its diagnostic potency was determined by ELISA [46].

### Protective potential of the prepared vaccine candidate

#### Cryptosporidium parvum oocyst shedding in feces of vaccinated infected and non-vaccinated infected mice

Details of *C*. *parvum* oocyst shedding in the feces of non-vaccinated infected and vaccinated infected mice are shown in Table-1. Oocyst shedding was detected using MZN staining in the two groups starting from day 3 PI in the non-vaccinated infected group and from day 5 PI in the vaccinated infected group. We observed a time-dependent reduction in oocyst shedding throughout the experimental period in both infected groups. A remarkable reduction occurred earlier (day 9 PI) in the vaccinated infected mice compared with non-vaccinated infected mice (t < 0.001), where a lower number of oocysts were shed from day 13 PI (t < 0.001). We did not detect any oocysts in the feces of non-vaccinated infected mice on day 18 PI, but they were present on days 19 and 20 PI and then absent again on the final day of the experiment (day 21 PI). On the other hand, *C*. *parvum* oocysts were eliminated completely in the vaccinated infected group on day 14 PI and were not observed for the remainder of the experiment. Furthermore, it is worth mentioning that a lower number of oocysts were recorded from the beginning of shedding and during the entire experimental period in the vaccinated infected group compared with the non-vaccinated infected group.

**Table-1:** *Cryptosporidium parvum* oocyst shedding in feces of infected and vaccinated mice groups

Animal groups/Days PI	*C. parvum* infected group	Vaccinated *C. parvum* infected group	t-value
Day 3	81.5 ± 0.563	0.0	144.829**
Day 4	85.167 ± 1.64	0.0	51.884**
Day 5	87.33 ± 1.15	70.67 ± 0.84	11.72**
Day 6	89.167 ± 1.37	79.667 ± 0.67	6.212**
Day 7	92.667 ± 2.23	83.833 ± 1.14	3.527*
Day 8	95.83 ± 1.14	89.5 ± 0.43	5.21*
Day 9	98.667 ± 0.85	56.167 ± 0.48	43.86**
Day 10	100.0 ± 0.86	42.0 ± 1.53	33.12**
Day 11	105.167 ± 1.17	16.833 ± 0.48	70.077**
Day 12	106.8 ± 1.01	13.83 ± 1.66	47.778**
Day 13	98.83 ± 0.4	1.0 ± 0.37	180.296**
Day 14	97.66 ± 0.61	0.0	158.9**
Day 15	93.5 ± 1.38	0.0	67.536**
Day 16	32.5 ± 0.85	0.0	38.391**
Day 17	13.0 ± 0.52	0.0	25.174**
Day 18	0.0	0.0	-
Day 19	17.5 ± 0.67	0.0	26.087**
Day 20	4.33 ± 0.55	0.0	7.769*
Day 21	0.0	0.0	-

Values are expressed as mean ± standard error.

* t < 0.01=Statistically significant.

** t < 0.001=High statistical significance. *C. parvum*=*Cryptosporidium parvum*, PI=Post infection

#### Humoral immune response

**Figure-2:**
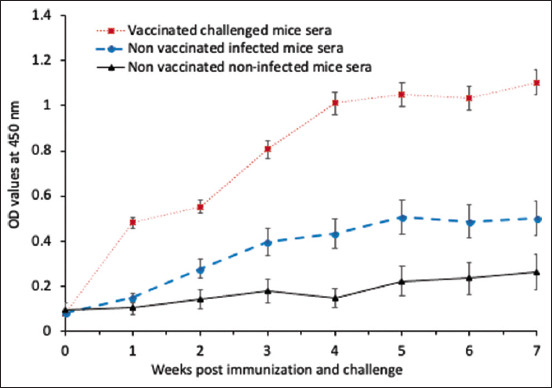
Immunoglobulin G antibody levels measured by enzyme-linked immunosorbent assay in vaccinated and non-vaccinated mice sera. Bars represent standard error.

As depicted in Figure-2, mice vaccinated with the purified fraction showed a stronger antibody response than the non-vaccinated groups (p < 0.05). The specific-IgG level against the purified fraction gradually developed 1 week after the first vaccination dose and then increased to record OD values (mean ± standard error: 0.553 ± 0.02) 2 weeks after the first vaccination dose, reaching an OD value of 1.011 ± 0.021 at 4 weeks after the first vaccination dose. After the challenge, the specific-IgG levels in the vaccinated infected mice were significantly higher than those in the non-vaccinated infected mice at all intervals of the experiment, starting at 1 week PI and peaking at 3 weeks PI (1.1034 ± 0.018).

#### Cytokine profiles

Tables-2–5 show IFN-γ, IL-10, IL-12, and IL-15 concentrations, respectively, in non-vaccinated non-infected, non-vaccinated infected, and vaccinated infected mice for the 7 weeks of the experiment. It must be mentioned that the *C. parvum* challenge was performed in non-vaccinated infected and vaccinated infected groups 4 weeks after the first vaccination.

**Table-2:** Interferon gamma concentrations in non-vaccinated non-infected, non-vaccinated *C. parvum* infected, and vaccinated infected mice groups

Animal groups/weeks	Non-vaccinated non-infected	Non-vaccinated *C. parvum*-infected	Vaccinated *C. parvum*-infected	F-value
1^st^ week	203.33 ± 10.93	196 ± 3.06	216.3 ± 2.31	2.364^NS^
2^nd^ week	204.67 ± 7.86	208.67 ± 5.7	221.97 ± 1.97	2.509^NS^
3^rd^ week	194.33 ± 2.33^b^	206.33 ± 9.4^b^	264.8 ± 1.27^a^	44.643**
4^th^ week	191.67 ± 1.6^b^	198 ± 1.53^b^	271.47 ± 4.5^a^	231.37**
5^th^ week (1^st^ week PI)	203.33 ± 3.33^c^	293.3 ± 2.17^b^	305.3 ± 0.35^a^	583.891**
6^th^ week	206.33 ± 8.76^b^	296.8 ± 2.23^a^	298.67 ± 2.29^a^	96.024**
7^th^ week	210 ± 1.34^c^	299.67 ± 0.88^a^	231.67 ± 6.1^b^	169.718**

*C. parvum*=*Cryptosporidium parvum*, NS=Non-significant, PI=Post infection. Values are expressed as mean ± standard error.

** p < 0.001=High statistical significance. Means within the same row with different superscripts are significantly different

**Table-3:** Interlukin-10 concentrations in non-vaccinated non-infected, non-vaccinated *C. parvum* infected, and vaccinated infected mice groups

Animal groups/weeks	Non-vaccinated non-infected	Non-vaccinated *C. parvum*-infected	Vaccinated *C. parvum*-infected	F-value
1^st^ week	74 ± 2.08^b^	70.33 ± 1.45^b^	90.67.3 ± 1.2^a^	44.662**
2^nd^ week	81.33 ± 1.86^b^	76 ± 2.31^b^	112.5 ± 0.87^a^	122.382**
3^rd^ week	83.67 ± 2.33^b^	82.67 ± 2.19^b^	120.67 ± 1.2^a^	120.6**
4^th^ week	84.37 ± 2.6^b^	84.33 ± 1.76^b^	121.83 ± 2.02^a^	124.41**
5^th^ week (1^st^ week PI)	74.33 ± 2.3^c^	95.92 ± 1.58^b^	125.33 ± 1.76^a^	177.726**
6^th^ week	76.67 ± 2.03^c^	106.67 ± 1.2^b^	131.33 ± 0.88^a^	355.018**
7^th^ week	73.83 ± 1.74^c^	125.17 ± 1.59^a^	102.5 ± 1.32^b^	271.757**

*C. parvum*=*Cryptosporidium parvum*, PI=Post infection. Values are expressed as mean ± standard error.

** p < 0.001=High statistical significance. Means within the same row with different superscripts are significantly different

**Table-4:** Interleukin-12 concentrations in non-vaccinated non-infected, non-vaccinated *C. parvum* infected, and vaccinated infected mice groups

Animal groups/weeks	Non-vaccinated non-infected	Non-vaccinated *C. parvum*-infected	Vaccinated *C. parvum*-infected	F-value
1^st^ week	82.5 ± 1.44	85 ± 2.52	87.33 ± 1.2	1.777^NS^
2^nd^ week	83.67 ± 2.73^b^	82 ± 1^b^	101.67 ± 4.41^a^	12.793*
3^rd^ week	85.67 ± 1.2^b^	84 ± 1.1^b^	143 ± 1.53^a^	708.581**
4^th^ week	85.97 ± 0.98^b^	84.37 ± 0.69^b^	144.3 ± 1.61^a^	867.678**
5^th^ week (1^st^ week PI)	83.33 ± 2.85^c^	142.17 ± 1.48^b^	156.17 ± 2.49^a^	271.577**
6^th^ week	82 ± 1.53^c^	156.17 ± 0.73^b^	165.67 ± 2.6^a^	653.138**
7^th^ week	79.5 ± 0.29^c^	160.83 ± 0.44^a^	125 ± 2.89^b^	578.868**

*C. parvum*=*Cryptosporidium parvum*, NS=Non-significant, PI=Post infection. Values are expressed as mean ± standard error.

* p < 0.05=Statistically significance.

** p < 0.001=High statistical significance. Means within the same row with different superscripts are significantly different

**Table-5:** Interlukin-15 concentrations in non-vaccinated non-infected, non-vaccinated *C. parvum* infected, and vaccinated infected mice groups

Animal groups/weeks	Non-vaccinated non-infected	Non-vaccinated *C. parvum*-infected	Vaccinated *C. parvum*-infected	F-value
1^st^ week	153.5 ± 2.84^b^	148.33 ± 2.19^b^	182 ± 1.53^a^	64.905**
2^nd^ week	154.5 ± 2.47^b^	152.83 ± 1.74^b^	187.5 ± 1.44^a^	102.442**
3^rd^ week	149.33 ± 1.76^b^	146.87 ± 1.16^b^	189 ± 0.58^a^	349.777**
4^th^ week	151.53 ± 0.87^b^	149.3 ± 0.65^b^	191.43 ± 0.86^a^	874.134**
5^th^ week (1^st^ week PI)	151.5 ± 0.87^b^	151.17 ± 0.73^b^	200 ± 0.58^a^	1470.121**
6^th^ week	152 ± 1.15^c^	198.17 ± 0.44^b^	231.33 ± 0.88^a^	2065.699**
7^th^ week	149.67 ± 0.88^c^	237 ± 1.53^a^	203.83 ± 0.73^b^	1602.298**

*C. parvum*=*Cryptosporidium parvum*, PI=Post infection, Values are expressed as mean ± standard error.

** p < 0.001=High statistical significance. Means within the same row with different superscripts are significantly different

There were no significant differences in IFN-γ concentration (Table-2) between the three groups in the 1^st^ and 2^nd^ weeks. Vaccinated infected mice showed a significant increase (p < 0.001) in IFN-γ concentrations in the 3^rd^ and 4^th^ weeks. The introduction of *C. parvum* infection in the 5^th^ week as a factor led to significantly increased levels of IFN-γ in non-vaccinated infected mice (p < 0.001) compared with non-vaccinated non-infected mice, but the level was still lower than that of the vaccinated infected mice (p < 0.001), which showed increased levels following the parasite challenge. In the 6^th^ week, the non-vaccinated infected and vaccinated infected mice had statistically similar IFN-γ levels. There was a significant reduction (p < 0.001) in IFN-γ levels in vaccinated infected mice in the 7^th^ week, while the levels in non-vaccinated infected mice were still increasing (p < 0.001).

Regarding IL-10 concentrations (Table-3), the vaccinated infected group showed a significant early increase (p < 0.001) from the 1^st^ week post first vaccination dose compared with the other two groups, which showed similar levels until the 5^th^ week. In the 5^th^ and 6^th^ weeks, the non-vaccinated infected mice showed increased IL-10 levels compared with non-vaccinated non-infected mice, but the level was still lower than the vaccinated mice (p < 0.001). In the 7^th^ week, there was a reduction in IL-10 levels in vaccinated infected mice (p < 0.001), but the levels in non-vaccinated infected mice were still increasing (p < 0.001).

IL-12 concentrations (Table-4) were statistically similar in all three examined groups in the 1^st^ week. The vaccinated infected mice showed increased IL-12 levels compared with the other two groups in the 2^nd^ (p < 0.05), 3^rd^ (p < 0.001), and 4^th^ (p < 0.001) weeks. The parasite challenge raised the IL-12 levels (p < 0.001) in the non-vaccinated infected mice and the vaccinated infected mice to levels higher than those in the non-vaccinated non-infected mice in the 5^th^ and 6^th^ weeks, with the levels in the vaccinated infected mice being the highest. In the 7^th^ week, IL-12 levels declined significantly (p < 0.001) in the vaccinated infected mice compared with the non-vaccinated infected mice.

Concerning IL-15 concentrations (Table-5), the vaccinated infected group showed an early increase (p < 0.001) from the 1^st^ week post-first vaccination dose compared with the other two groups, which were similar until the 6^th^ week. In the 6^th^ week, the non-vaccinated infected mice showed a greater increase in IL-15 concentrations than the non-vaccinated non-infected mice. However, the levels were still lower than the vaccinated infected mice (p < 0.001). In the 7^th^ week, there was a significant reduction (p < 0.001) in IL-15 levels in the vaccinated infected mice, and the levels in the non-vaccinated infected mice were still increasing (p < 0.001).

#### Histopathological findings in ileal and hepatic tissue

Ileal tissue

**Figure-3:**
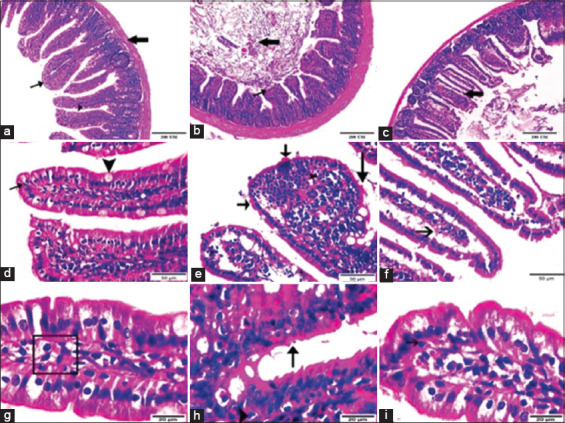
Photomicrographs displaying the histopathological changes in ileal tissue sections between studied mice groups; (a, d and g) Ileal sections of non-infected non-vaccinated group presented the normal histological structure of ileum as follows: lining epithelium of simple columnar absorptive (thin arrow) with goblet cells in between (arrowhead), lamina propria of dense irregular connective tissue (cube), simple tubular intestinal gland (circle), and muscular layer organized as inner circular and outer longitudinal of smooth muscle cells (thick arrow). (b, e and h) Ileal sections of *Cryptosporidium parvum*-infected group highlighted severe degeneration of ileal tissue as shortening of villi, sloughing and erosion of lining epithelium, aggregated *Cryptosporidium parvum* developmental stages (arrows), as well as infiltration of inflammatory cells (arrowhead). (c, f and i) Ileal sections of vaccinated infected mice group revealed less alterations in tissue structure as most villi appeared in normal architecture except some (c) with sloughing and erosions of epithelial cells, (f) vacuolations in lamina propria, (i) and few *Cryptosporidium parvum* developmental stages. (Hematoxylin and Eosin Stain, Magnification Power; 100×, 400×, 100×, respectively, Scale bar; 200 μm, 50 μm, 20 μm, respectively).

The histopathological changes in ileal tissue sections for each group are presented in Figure-3. Ileal sections of non-vaccinated non-infected mice showed a normal histological structure. Ileal sections of non-vaccinated *C*. *parvum*-infected mice showed severe degeneration of ileal tissue with villi shortening, sloughing and erosion of the lining epithelium, aggregations of *C*. *parvum* developmental stages, and inflammatory cell infiltration. Sections of ileal tissue from vaccinated infected mice had fewer alterations in tissue structure compared with non-vaccinated infected mice. Most villi showed normal architecture, except some with sloughing and erosion of epithelial cells. Additionally, there were vacuolations in the lamina propria and a few *C*. *parvum* developmental stages.

Hepatic tissue

**Figure-4:**
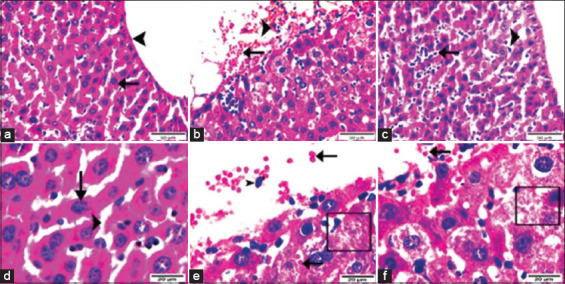
Photomicrographs demonstrated the histopathological alterations in liver tissue sections between experimented mice groups; (a and d) Liver sections of non-infected non-vaccinated group detailed the normal hepatic plates composition: (a) Central vein area with its endothelial lining of simple squamous epithelium (arrowhead), hepatocytes organized in regular cords (arrow), (d) nucleus of hepatocytes presented centrally in a light vesicular form (arrow). Notice hepatic sinusoids between cords (arrowhead). (b and e) Liver sections of *Cryptosporidium parvum*-infected group highlighted intense destructive hepatic tissue with severe manifestation of *Cryptosporidium parvum* developmental stages (arrows) and inflammatory cells (arrowhead) alongside necrotic areas (cube). (c and f) Liver sections of vaccinated infected mice group exposed few changes in hepatic tissue structure, (c) vacuolations (arrowhead) and aggregated inflammatory cells in hepatic sinusoids (arrow), and (f) limited necrotic areas (cube) and few *Cryptosporidium parvum* developmental stages. (Hematoxylin and Eosin Stain, Magnification Power; 100×, 400×, respectively, Scale bar; 200).

The histopathological changes in hepatic tissue sections for each group are presented in Figure-4. Hepatic sections of non-vaccinated non-infected mice showed normal hepatic plate composition. Sections of hepatic tissue from the non-vaccinated *C*. *parvum*-infected mice showed extensive tissue destruction with a severe manifestation of *C*. *parvum* developmental stages and inflammatory cells alongside necrotic areas. Sections of hepatic tissue from the vaccinated infected group showed fewer changes in hepatic tissue structure, including vacuolations and aggregated inflammatory cells in hepatic sinusoids, limited necrotic areas, and few *C*. *parvum* developmental stages.

#### Count of C. parvum oocysts and developmental stages in ileal and hepatic sections

Numerous *C*. *parvum* developmental stages were present in the ileal and hepatic sections of infected and vaccinated mice (Table-6). The parasite was found more often in the ileum than the liver (p < 0.001), and there was a statistically significant decrease (p < 0.05) in its count in the vaccinated infected group compared with the non-vaccinated infected group.

**Table-6:** *Cryptosporidium parvum* oocysts and developmental stages count in ileum and liver of non-vaccinated infected and vaccinated infected groups

Parameter/organ	Non-vaccinated *C. parvum* infected group	Vaccinated *C. parvum* infected group	t-value
Oocysts and developmental stages count			
Ileum	506.83 ± 33.264*	81.67 ± 2.590	12.743*
Liver	229.83 ± 15.296*	22.667 ± 2.486	13.369*

Values are expressed as mean ± standard error.

* t<0.05: statistically significant

## Discussion

Animal models have been used to study different *Cryptosporidium* species, including their pathogenicity and host specificity, as well as to evaluate vaccines and drug treatments. Mice are used to study parasitic diseases because they have a strong reproductive ability, only slight individual differences, high genetic stability, and susceptibility to various pathogens [47]. In this study, we used mice to examine the protective potential of a *C. parvum* vaccine candidate prepared from affinity-purified antigen by assessing fecal oocyst shedding, IgG response, cytokine levels, histopathological changes in the ileum and liver, and the number of parasite developmental stages in ileal and hepatic tissues.

We detected *Cryptosporidium* spp. in preweaned buffalo calves, and we used PCR to confirm the species as *C. parvum*. This parasite has been detected in cattle [48], buffaloes [49], sheep and goats [50], horses and donkeys [51], and camels [52], as well as in humans [53]. In addition, *C. parvum* was found to be the most dominant and pathogenic species in farm animals [6] and has been detected worldwide in both farm animals and humans [54]. There are variations in the recorded prevalence of *C. parvum*, which may be attributed to differences in animal ages, locality, rearing systems, stress, breeding techniques, and the level of hygienic measures applied [55]. Here, we report a cryptosporidiosis infection rate of 75% in the feces of newborn diarrheic buffalo calves, which is correlated with the reported prevalence of *C. parvum* in preweaned calves of 3.4%–96.6% [5, 56–58]. The age at sampling is crucial because calves aged <6 weeks are most likely to shed *C. parvum*, while other animals might shed other (nonpathogenic) species of *Cryptosporidium* [5].

In this study, we observed a prolonged prepatent period and decreased oocyst shedding in the vaccinated infected mice compared with the non-vaccinated infected mice (t < 0.001), suggesting that the affinity-purified vaccine candidate was potent and provided effective protection against *C. parvum* infection. This correlates with the results reported by Liu *et al*. [28] for mice immunization with a *C. parvum* divalent peptide vaccine candidate. However, the higher potency of our prepared vaccine compared with those evaluated by Liu *et al*. [28] might be due to the difference in mice age as they used older mice and the hypothesis that neonatal animals are necessary for a successful vaccination [59] in addition to a different vaccine preparation method. Oocyst shedding patterns and counting the number of oocysts have been previously evaluated [60–64]. The percentage of oocyst shedding reduction in the feces of immunized infected mice on day 12 PI (the peak of oocyst shedding in the non-vaccinated infected group) was 87.05%, which was better than previous reports on DNA vaccines that reduced oocyst shedding by 54.5%–77.5% after challenge with *C. parvum* [65, 66].

In our study, IgG levels in the non-vaccinated infected mice were significantly higher than those of non-vaccinated non-infected mice at all weeks PI. This supports the assumption that *C. parvum*-specific antibodies are activated during infection and can be interpreted as a marker for cryptosporidiosis [27]. In our study, the IgG levels after challenge in the vaccinated infected mice were significantly higher than in the infected mice at all intervals starting from 1 week PI and peaked at 3 weeks PI. Yu, *et al*. [67] also recorded a similar result when investigating Cp12 and Cp21 antigens as vaccine candidates in mice. The role of secretory and serum antibodies in opposing cryptosporidiosis was proved by the reduction in oocyst excretion with the increased production of antibodies. Our findings in mice agreed with those of Yu and Lee [68] and other studies on human infections, suggesting a correlation between IgG response and the extent of infection [27]. Furthermore, this has also been demonstrated in lambs [69] and calves [70]. In addition, El Shazly *et al*. [71] recorded a significant correlation between IgG response and the *C. parvum* oocyst count in infected children.

Regarding cytokine profiles, we found that the vaccinated mice showed activation of cytokines IL-10 and IL-15 (p < 0.001) from the 1^st^ week post-first vaccination dose. Interleukin-12 levels increased from the 2^nd^ week post-first vaccination dose (p < 0.05), while IFN-γ increased from the third. Although IFN-γ was assumed to be an important mediator of innate and acquired immune responses against cryptosporidiosis in murine models [72] and is critical for memory response, other mechanisms may play a larger role in acute infection [31]. After the challenge, all tested cytokines significantly increased (p < 0.001) in non-vaccinated infected and vaccinated infected mice compared with non-vaccinated non-infected mice and were higher in vaccinated mice until the 7^th^ week. The high levels of IFN-γ and IL-12 recorded in our study after the challenge correlate with the results obtained by McNair and Mead [73], Abouel-Nour *et al*. [74], Laurent and Lacroix-Lamandé [13], and Aboelsoued *et al*. [58, 75]. It has been suggested that these high amounts of cytokines were produced to clear the parasite from the intestinal epithelium [76]. In addition, these high levels may be present because IFN-γ and IL-12 are primarily released by T lymphocytes and NK cells in response to stimuli propagated by the excysted sporozoites invading intestinal epithelium, where the parasite completes its life cycle [5]. The increased levels of IL-10 observed in our study agree with the findings of Lacroix *et al*. [76] and Gaber *et al*. [14], who observed increased IL-10 levels during *C. parvum* infection. El-Wakil *et al*. [77] and Chang *et al*. [78] demonstrated that cytokine levels played an important role in controlling cryptosporidiosis. In the 7^th^ week of our study, cytokine levels started to decrease significantly (p < 0.001) in the vaccinated infected group (in which no oocysts appeared in feces the whole week) compared with the non-vaccinated infected group. This might indicate that the protective effect of the prepared vaccine led to elevated cytokine concentrations before and after infection, which, in turn, reduced oocyst shedding to none before returning cytokine concentrations to normal levels.

The ileal sections of *C. parvum*-infected mice showed severe tissue degeneration, shortened villi, sloughing, and erosion of the lining epithelium, aggregations of *Cryptosporidium* oocysts, and inflammatory cell infiltrates. These findings were also observed by Al-Mathal and Alsalem [61], Abouel-Nour *et al*. [79], Aboelsoued *et al*. [63, 64], and Shahbazi *et al*. [80] in mice experimentally infected with *Cryptosporidium* and may be due to impaired intestinal absorption, increased paracellular permeability, and impaired barrier function after infection [81, 82]. Mead [20] stated that injury to the intestinal epithelial architecture due to infection and inflammation could alter the tight junctions between epithelial cells and induce innate inflammatory responses. The hepatic sections of mice in the *C. parvum*-infected group showed extreme destruction of hepatic tissue with a severe manifestation of *Cryptosporidium* oocysts and inflammatory cells alongside necrotic areas. These observations agree with those of Abdou *et al*. [83] and Elmahallawy *et al*. [84] in *C*. parvum*-*experimentally infected mice. Sections of ileal and hepatic tissue in the vaccinated infected group in our study showed few structural changes and a low number of *C. parvum* developmental stages. This might be due to the reduced severity of infection, a longer prepatent period, and lower parasite loads in feces in response to immunization, which might also cause a slight pathogenic effect in infected tissues.

Our findings on the endogenous developmental stages of *C. parvum* on day 10 PI indicate that the ileum was more parasitized than the liver and correlate with Perrucci *et al*. [60], Al-Mathal and Alsalem [85], and Asadpour *et al*. [62]. Our results also showed that vaccination of mice resulted in a lower number of oocysts in ileal and hepatic tissues (p < 0.05), suggesting that our prepared vaccine candidate reduced the *C. parvum* load in tissues.

## Conclusion

As a vaccine candidate, the affinity-purified *C. parvum* oocyst antigen successfully elicited protective cellular and humoral responses against mice cryptosporidiosis, resulting in a significant reduction in *C. parvum* oocyst shedding, histopathological changes in the ileum and liver, and the number of parasite developmental stages in tissues. Our findings indicate that the prepared vaccine could be a promising candidate for protection against cryptosporidiosis. Further studies on this vaccine candidate using other animal models and animal field trials should be performed to evaluate its potency and prepare it for large-scale production.

## Authors’ Contributions

DA and KNAM: Study design and conceptualization. DA, NIT and HHAMA: Data collection, methodology, data validation and interpretation, original manuscript drafting, and reviewing. DA: Project administration and statistical analysis. DA, HHAMA, KNAM, SEH, and NIT: Writing – review and editing. All authors have read and approved the final manuscript.

## References

[1] Checkley W, White A.C, Jaganath D, Arrowood M.J, Chalmers R.M, Chen X.M, Fayer R, Griffiths J.K, Guerrant R.L, Hedstrom L, Huston C.D, Kotloff K.L, Kang G, Mead J.R, Miller M, Petri W.P, Priest J.W, Roos D.S, Striepen B, Thompson R.C.A, Ward H.D, Van Voorhis W.A, Xiao L, Zhu G, Houpt E.R (2015). A review of the global burden, novel diagnostics, therapeutics, and vaccine targets for *Cryptosporidium*. Lancet Infect. Dis.

[2] Shirley D.A, Moonah S.N, Kotloff K.L (2012). Burden of disease from cryptosporidiosis. Curr. Opin. Infect. Dis.

[3] Kotloff K.L, Nataro J.P, Blackwelder W.C, Narsin D, Farag T.H, Panchalingam S, Wu Y, Sow S.O, Sur D, Breiman R.F, Faruque A.S, Zaidi A.K, Saha D, Alonso P.L, Tamboura B, Sanogo D, Onwuchekwa U, Manna B, Ramamurthy T, Kanungo S, Ochieng J.B, Omore R, Oundo J.O, Hossain A, Das S.K, Ahmed S, Qureshi S, Quadri F, Adegbola R.A, Antonio M, Hossain M.J, Akinsola A, Mandomando I, Nhampossa T, Acácio S, Biswas K, O'Reilly C.E, Mintz E.D, Berkeley L.Y, Muhsen K, Sommerfelt H, Robins-Browne R.M, Levine M.M (2013). Burden and aetiology of diarrhoeal disease in infants and young children in developing countries (the global enteric multicenter study, GEMS):A prospective, case-control study. Lancet.

[4] Bowman D.D, Georgi J.R (2014). Georgis'Parasitology for Veterinarians.

[5] Thomson S, Hamilton C.A, Hope J.C, Katzer F, Mabbott N.A, Morrison L.J, Innes E.A (2017). Bovine cryptosporidiosis:Impact, host-parasite interaction and control strategies. Vet. Res.

[6] Tomazic M.L, Garro C.J, Schnittger L, Florin-Christensen M, Schnittger L (2018). Cryptosporidium. Parasitic Protozoa of Farm Animals and Pets.

[7] Adkins P.R.F (2022). Cryptosporidiosis. Vet. Clin. North Am. Food Anim. Pract.

[8] Pinto D.J, Vinayak S (2021). *Cryptosporidium*:Host-parasite interactions and pathogenesis. Curr. Clin. Microbiol. Rep.

[9] Innes E.A, Chalmers R.M, Wells B, Pawlowic M.C (2020). A one health approach to tackle cryptosporidiosis. Trends Parasitol.

[10] Da Silva D.R.R, de Oliveira C.B, Marta B.B.F, Baptista C.B, Bottaro M.C, Bresciani K.D (2021). Vaccine development for cryptosporidiosis:Systematic review. Res. Soc. Dev.

[11] Ludington J.G, Ward H.D (2015). Systemic and mucosal immune responses to *Cryptosporidium*-vaccine development. Curr. Trop. Med. Rep.

[12] Kasper L.H, Buzoni-Gatel D (2001). Ups and downs of mucosal cellular immunity against protozoan parasites. Infect. Immun.

[13] Laurent F, Lacroix-Lamandé S (2017). Innate immune responses play a key role in controlling infection of the intestinal epithelium by *Cryptosporidium*. Int. J. Parasitol.

[14] Gaber M, Galal L.A.A, Hassan D, Badary D.M, Mohamed I.M (2020). Evidences of brain and lung invasion of a local water *Cryptosporidium parvum* isolate in comparison to Iowa strain:serological and immunohistochemical cytokine evaluation. Ann. Parasitol.

[15] Riggs M.W (2002). Recent advances in cryptosporidiosis:The immune response. Microbes Infect.

[16] Deng M, Rutherford M.S, Abrahamsen M.S (2004). Host intestinal epithelial response to *Cryptosporidium parvum*. Adv. Drug Deliv. Rev.

[17] Korbel D.S, Barakat F.M, Di Santo J.P, McDonald V (2011). CD4+T cells are not essential for control of early acute *Cryptosporidium parvum* infection in neonatal mice. Infect. Immunol.

[18] Hunter C.A, Sibley L.D (2012). Modulation of innate immunity by *Toxoplasma Gondii* virulence effectors. Nat. Rev. Microbiol.

[19] McDonald V, Korbel D.S, Barakat F.M, Choudhry N, Petry F (2013). Innate immune responses against *Cryptosporidium parvum* infection. Parasite Immunol.

[20] Mead J.R (2014). Prospects for immunotherapy and vaccines against Cryptosporidium. Hum. Vaccine Immunother.

[21] Cortez V.S, Colonna M (2016). Diversity and function of group 1 innate lymphoid cells. Immunol. Lett.

[22] Partida-Rodríguez O, Serrano-Vázquez A, Nieves-Ramírez M.E, Moran P, Rojas L, Portillo T, González E, Hernández E, Finlay B.B, Ximenez C (2017). Human intestinal microbiota:Interaction between parasites and the host immune response. Arch. Med. Res.

[23] Ivanova D.L, Denton S.L, Fettel K.D, Sondgeroth K.S, Munoz Gutierrez J, Bangoura B, Dunay I.R, Gigley J.P (2019). Innate lymphoid cells in protection, pathology, and adaptive immunity during apicomplexan infection. Front. Immunol.

[24] Perera P.Y, Lichy J.H, Waldmann T.A, Perera L.P (2012). The role of interleukin-15 in inflammation and immune responses to infection:implications for its therapeutic use. Microbes Infect.

[25] Dann S.M, Wang H.C, Gambarin K.J, Actor J.K, Robinson P, Lewis D.E, Caillat-Zucman S, White A.C (2005). Interleukin-15 activates human natural killer cells to clear the intestinal protozoan *Cryptosporidium*. J. Infect. Dis.

[26] Lang C, Groß U, Lüder C.G.K (2007). Subversion of innate and adaptive immune responses by *Toxoplasma gondii*. Parasitol. Res.

[27] Jakobi V, Petry F (2008). Humoral immune response in IL-12 and IFN-γ deficient mice after infection with *Cryptosporidium parvum*. Parasite Immunol.

[28] Liu K, Zai D, Zhang D, Wei Q, Han G, Gao H, Huang B (2010). Divalent Cp15-23 vaccine enhances immune responses and protection against *Cryptosporidium parvum* infection. Parasite Immunol.

[29] Akdis M, Aab A, Altunbulakli C, Azkur K, Costa R.A, Crameri R, Duan S, Eiwegger T, Eljaszewicz A, Ferstl R, Frei R, Garbani M, Globinska A, Hess L, Huitema C, Kubo Komlosi Z, Konieczna P, Kovacs N, Kucuksezer U.C, Meyer N, Morita H, Olzhausen J, O'Mahony L, Pezer M, Prati M, Rebane A, Rhyner C, Rinaldi A, Sokolowska M, Stanic B, Sugita K, Treis A, van de Veen W, Wanke K, Wawrzyniak M, Wawrzyniak P, Wirz O.F, Zakzuk J.S, Akdis C.A (2016). Interleukins (from IL-1 to IL-38), interferons, transforming growth factor b, and TNF- :receptors, functions, and roles in diseases. J. Allergy Clin. Immunol.

[30] Moss D.M, Chappell C.L, Okhuysen P.C, DuPont H.L, Arrowood M.J, Hightower A.W, Lammie P.J (1998). The antibody response to 27-, 17-,and 15-kDa *Cryptosporidium* antigens following experimental infection in humans. J. Infect. Dis.

[31] Pantenburg B, Dann S.M, Wang H.C, Robinson P, Castellanos-Gonzalez A, Lewis D.E, White A.C (2008). Intestinal immune response to human *Cryptosporidium* spp. infection. Infect. Immun.

[32] Kaufmann J (1996). Parasitic Infections of Domestic Animals:A Diagnostic Manual.

[33] Henriksen S.A, Pohlenz J.F (1981). Staining of Cryptosporidia by a modified Ziehl-Neelsen technique. Acta Vet. Scand.

[34] Current W.L, Reese N.C (1986). A comparison of endogenous development of three isolates of *Cryptosporidium* in suckling mice. J. Protozool.

[35] Feltus D.C, Giddings C.W, Schneck B.L, Monson T, Warshauer D, McEvoy J.M (2006). Evidence supporting zoonotic transmission of *Cryptosporidium* spp. in Wisconsin. J. Clin. Microbiol.

[36] Thompson J.D, Higgins D.G, Gibson T.J (1994). CLUSTAL W:Improving the sensitivity of progressive multiple sequence alignment through sequence weighting, position-specific gap penalties and weight matrix choice. Nucleic Acids Res.

[37] Tamura K, Stecher G, Peterson D, Filipski A, Kumar S (2013). MEGA6:Molecular evolutionary genetics analysis version 6.0. Mol. Biol. Evol.

[38] Del Coco V.F, Córdoba M.A, Sidoti A, Santín M, Drut R, Basualdo J.A (2012). Experimental infection with *Cryptosporidium parvum* IIaA21G1R1 subtype in immunosuppressed mice. Vet. Parasitol.

[39] Current W.L, Dubey J.P, Speer C,A, Fayer R (1990). Techniques and laboratory maintenance of *Cryptosporidium*. Cryptosporidiosis of Man and Animals.

[40] Kaushik K, Khurana S, Wanchu A, Malla N (2009). Serum immunoglobulin G, M and A response to *Cryptosporidium parvum* in *Cryptosporidium*-HIV co-infected patients. BMC Infect. Dis.

[41] Fagbemi B.O, Obarisiaghon I.O, Mbuh J.V (1995). Detection of circulating antigen in sera of *Fasciola gigantica* infected cattle with antibodies reactive with a *Fasciola*-specific 88 kDa antigen. Vet. Parasitol.

[42] Lowry O.H, Rosebrough N.J, Farr A.B, Randall R.J (1951). Protein measurement with the folin-phenol reagent. J. Biol. Chem.

[43] Ortolani E (2000). Standardization of the modified Ziehl-Neelsen technique to stain oocysts of *Cryptosporidium* spp. Rev. Bras. Parasitol.

[44] Priest J.W, Kwon J.P, Moss D.M, Roberts J.M, Arrowood M.J, Dworkin M.S, Juranek D.D, Lammie P.J (1999). Detection by enzyme immunoassay of serum immunoglobulin G antibodies that recognize specific *Cryptosporidium parvum* antigen. J. Clin. Microbiol.

[45] Bancroft J.D, Stevens A (2013). A Theory and Practice of Histological Techniques.

[46] Aboelsoued D, Hendawy S, Abdullah H.H.A, Megeed K.N.A, El Hakim A.E, Hassan S.E, Toaleb N.I (2022). Diagnosis of cryptosporidiosis using affinity-purified antigen. Egypt. J. Vet. Sci.

[47] Huang Y, Song Y, You Y, Mi R, Han H, Gong H, Chen Z, Liu Y (2021). Development of an immunocompetent mouse model susceptible to *Cryptosporidium tyzzeri* infection. Parasite Immunol.

[48] Mammeri M, Chevillot A, Chenafi I, Thomas M, Julien C, Vallée I, Polack B, Follet J, Adjou K.T (2019). Molecular characterization of *Cryptosporidium* isolates from diarrheal dairy calves in France. Vet. Parasitol. Reg. Stud. Reports.

[49] Ibrahim M.A, Abdel-Ghany A.E, Abdel-Latef G.K, Abdel-Aziz S.A, Aboelhadid S.M (2016). Epidemiology and public health significance of *Cryptosporidium* isolated from cattle, buffaloes, and humans in Egypt. Parasitol. Res.

[50] Baroudi D, Hakem A, Adamu H, Amer S, Khelef D, Adjou K, Dahmani H, Chen X, Roellig D, Feng Y, Xiao L (2018). Zoonotic *Cryptosporidium* species and subtypes in lambs and goat kids in Algeria. Parasit. Vectors.

[51] Li F, Su J, Chahan B, Guo Q, Wang T, Yu Z, Guo Y, Li N, Feng Y, Xiao L (2019). Different distribution of *Cryptosporidium* species between horses and donkeys. Infect. Genet. Evol.

[52] El-Alfy E.S, Abu-Elwafa S, Abbas I, Al-Araby M, Al-Kappany Y, Umeda K, Nishikawa Y (2019). Molecular screening approach to identify protozoan and trichostrongylid parasites infecting one-humped camels (*Camelus dromedarius*). Acta Trop.

[53] Guo Y, Ryan U, Feng Y, Xiao L (2022). Emergence of zoonotic *Cryptosporidium parvum* in China. Trends Parasitol.

[54] Ryan U, Zahedi A, Feng Y, Xiao L (2021). An update on zoonotic *Cryptosporidium* species and genotypes in humans. Animals (*Basel*).

[55] Kváč M, Kouba M, Vítovec J (2006). Age-related and housing-dependence of *Cryptosporidium* infection of calves from dairy and beef herds in South Bohemia, Czech Republic. Vet. Parasitol.

[56] Rieux A, Paraud C, Pors I, Chartier C (2013). Molecular characterization of *Cryptosporidium* isolates from pre-weaned calves in western France in relation to age. Vet. Parasitol.

[57] Essa S.H, Galila E.M, Abdelwahab M.G, Moustafa A.M, Hamouda F, El-Akabawy L (2014). Compare microscopy staining and polymerase chain reaction for diagnosis of *Cryptosporidium* infection among Frisian calves in Minufiya governorate. Benha Vet. Med. J.

[58] Aboelsoued D, Hendawy S.H.M, Abo-Aziza F.A.M, Abdel Megeed K.N (2020). Copro-microscopical and immunological diagnosis of cryptosporidiosis in Egyptian buffalo-calves with special reference to their cytokine profiles. J. Parasit. Dis.

[59] Perryman L.E, Kapil S.J, Jones M.L, Hunt E.L (1999). Protection of calves against cryptosporidiosis with immune bovine colostrum induced by a *Cryptosporidium parvum* recombinant protein. Vaccine.

[60] Perrucci S, Fichi G, Buggiani C, Rossi G, Flamini G (2006). Efficacy of mangiferin against *Cryptosporidium parvum* in a neonatal mouse model. Parasitol. Res.

[61] Al-Mathal E.M, Alsalem A.A (2012). Pomegranate (*Punica granatum*) peel is effective in a murine model of experimental *Cryptosporidium*
*parvum*. Exp. Parasitol.

[62] Asadpour M, Namazi F, Razavi S.M, Nazifi S (2018). Curcumin:A promising treatment for *Cryptosporidium parvum* infection in immunosuppressed BALB/c mice. Exp. Parasitol.

[63] Aboelsoued D, Abo-Aziza F.A.M, Mahmoud M.H, Megeed K.N.A, El Ezz N.M.T, Abu-Salem F.M (2019). Anticryptosporidial effect of pomegranate peels water extract in experimentally infected mice with special reference to some biochemical parameters and antioxidant activity. J. Parasit. Dis.

[64] Aboelsoued D, Shaapan R.M, Ekhateeb R.M.M, El-Nattat W.S, Elhameed M.F, Hammam A.M.M, Hammam A.M (2020). Therapeutic efficacy of ginger (*Zingiber officinale*), ginseng (*Panax ginseng*) and sage (*Salvia officinalis*) against *Cryptosporidium parvum* in experimentally infected mice. Egypt. J. Vet. Sci.

[65] Wang C, Luo J, Amer S, Guo Y, Hu Y, Lu Y, Wang H, Duan M, He H (2010). Multivalent DNA vaccine induces protective immune responses and enhanced resistance against *Cryptosporidium parvum* infection. Vaccine.

[66] Tian G, Yu S, Song J, Wu L, Wang H, Kong X, Zhang F, Chou X (2020). Development of DNA vaccine encoding *Cryptosporidium parvum* AOX and TSP6 genes and study on its immunoprotection effects. Prog. Vet. Med.

[67] Yu Q, Li J, Zhang X, Gong P, Zhang G, Li S, Wang H (2010). Induction of immune responses in mice by a DNA vaccine encoding *Cryptosporidium parvum* Cp12 and Cp21 and its effect against homologous oocyst challenge. Vet. Parasitol.

[68] Yu J.R, Lee S.U (2007). Time gap between oocyst shedding and antibody responses in mice infected with *Cryptosporidium parvum*. Korean J. Parasitol.

[69] Ortega-Mora L.M, Troncoso J.M, Rojo-Vázquez F.A, Gómez-Bautista M (1993). Serum antibody response in lambs naturally and experimentally infected with *Cryptosporidium parvum*. Vet. Parasitol.

[70] O'donogue P.J (1995). *Cryptosporidium* and cryptosporidiosis in man and animals. Int. J. Parasitol.

[71] El Shazly A.M, Soltan D.M, El-Sheikha H.M, Sadek G.S, Morsy A.T (2007). Correlation of ELISA copro-antigen and oocysts count to the severity of cryptosporidiosis parvum in children. J. Egypt. Soc. Parasitol.

[72] Leav B.A, Yoshida M, Rogers K, Cohen S, Godiwala N, Blumberg R.S, Ward H (2005). An early intestinal mucosal source of gamma interferon is associated with resistance to and control of *Cryptosporidium parvum* infection in mice. Infect. Immun.

[73] McNair N.N, Mead J.R (2013). CD4+effector and memory cell populations protect against *Cryptosporidium parvum* infection. Microbes Infect.

[74] Abouel-Nour M.F, EL-Shewehy D.M, Hamada S.F, Morsy T.A (2015). The efficacy of three medicinal plants:Garlic, ginger and mirazid and a chemical drug metronidazole against *Cryptosporidium parvum*. I-Immunological response. J. Egypt. Soc. Parasitol.

[75] Aboelsoued D, Toaleb N.I, Abdel Megeed K.N, Hassan S.E, Ibrahim S (2019). Cellular immune response and scanning electron microscopy in the evaluation of Moringa leaves aqueous extract effect on *Cryptosporidium parvum* in buffalo intestinal tissue explants. J. Parasit. Dis.

[76] Lacroix S, Mancassola R, Naciri M, Laurent F (2001). *Cryptosporidium parvum*-specific mucosal immune response in C57BL/6 neonatal and g-interferon-deficient mice:Role of tumor necrosis factor a in protection. Infect. Immun.

[77] El-Wakil E.S, Salem A.E, Al-Ghandour A.M.F (2021). Evaluation of possible prophylactic and therapeutic effect of mefloquine on experimental cryptosporidiosis in immunocompromised mice. J. Parasit. Dis.

[78] Chang L, Chen Y, Qian Kang J, Liu Z (2022). Detection of expression alteration of cytokines in the intestine of Balb/c mice infected with *Cryptosporidium parvum* using relative fluorescence quantitative PCR method. Pak. J. Zool.

[79] Abouel-Nour M.F, EL-Shewehy D.M, Hamada S.F, Morsy T.A (2016). The efficacy of three medicinal plants:Garlic, ginger and mirazid and a chemical drug metronidazole against *Cryptosporidium parvum*. II-Histological changes. J. Egypt. Soc. Parasitol.

[80] Shahbazi P, Nematollahi A, Arshadi S, Farhang H.H, Shahbazfar A.A (2021). The protective effect of *Artemisia spicigera* ethanolic extract against *Cryptosporidium parvum* infection in immunosuppressed mice. Iran. J. Parasitol.

[81] Klein P, Kleinová T, Volek Z, Simůnek J (2008). Effect of *Cryptosporidium parvum* infection on the absorptive capacity and paracellular permeability of the small intestine in neonatal calves. Vet. Parasitol.

[82] Di Genova B.M, Tonelli R.R (2016). Infection strategies of intestinal parasite pathogens and host cell responses. Front. Microbiol.

[83] Abdou A.G, Harba N.M, Afifi A.F, Elnaidany N.F (2013). Assessment of Cryptosporidium parvum infection in immunocompetent and immunocompromised mice and its role in triggering intestinal dysplasia. Int. J. Infect. Dis.

[84] Elmahallawy E.K, Elshopakey G.E, Saleh A.A, Agil A, El-Morsey A, M El-Shewehy D.M, Sad A.S, Yanai T, Abdo W (2020). S-Methylcysteine (SMC) ameliorates intestinal, hepatic, and splenic damage induced by *Cryptosporidium parvum* infection via targeting inflammatory modulators and oxidative stress in swiss albino mice. Biomedicines.

[85] Al-Mathal E.M, Alsalem A.A (2013). Pomegranate (*Punica granatum*) peel is effective in a murine model of experimental *Cryptosporidium parvum* ultrastructural studies of the ileum. Exp. Parasitol.

